# Quantification of (1→4)-β-d-Galactans in Compression Wood Using an Immuno-Dot Assay

**DOI:** 10.3390/plants4010029

**Published:** 2015-01-14

**Authors:** Ramesh R. Chavan, Leona M. Fahey, Philip J. Harris

**Affiliations:** School of Biological Sciences, The University of Auckland, Private Bag 92019, Auckland 1142, New Zealand; E-Mails: r.chavan@auckland.ac.nz (R.R.C.); lfah338@aucklanduni.ac.nz (L.M.F.)

**Keywords:** *Pinus radiata*, coniferous gymnosperms, compression wood, (1→4)-β-d-galactans, immuno-dot assay, LM5 monoclonal antibody, monosaccharide composition, delignification

## Abstract

Compression wood is a type of reaction wood formed on the underside of softwood stems when they are tilted from the vertical and on the underside of branches. Its quantification is still a matter of some scientific debate. We developed a new technique that has the potential to do this based on the higher proportions of (1→4)-β-d-galactans that occur in tracheid cell walls of compression wood. Wood was milled, partially delignified, and the non-cellulosic polysaccharides, including the (1→4)-β-d-galactans, extracted with 6 M sodium hydroxide. After neutralizing, the solution was serially diluted, and the (1→4)-β-d-galactans determined by an immuno-dot assay using the monoclonal antibody LM5, which specifically recognizes this polysaccharide. Spots were quantified using a dilution series of a commercially available (1→4)-β-d-galactan from lupin seeds. Using this method, compression and opposite woods from radiata pine (*Pinus* *radiata*) were easily distinguished based on the amounts of (1→4)-β-d-galactans extracted. The non-cellulosic polysaccharides in the milled wood samples were also hydrolysed using 2 M trifluoroacetic acid followed by the separation and quantification of the released neutral monosaccharides by high performance anion exchange chromatography. This confirmed that the compression woods contained higher proportions of galactose-containing polysaccharides than the opposite woods.

## 1. Introduction

Compression wood is a type of reaction wood formed by coniferous gymnosperms (softwoods) on the underside of stems that have been tilted from the vertical [1,2,3,4,5,6]. This type of wood is important to the tree because it restores normal, vertical growth to the stem. It can also be formed on the underside of branches where it has a role in maintaining the angle of the branch to the stem. However, it is regarded as a defect in the timber industry as, on drying, it exhibits higher longitudinal shrinkage than normal wood or wood on the opposite side of the stem (opposite wood) and contributes to warping. It is also more difficult to work with and pulp than normal or opposite wood [5,6]. Compression wood is often darker in colour than normal and opposite wood, but the chemical basis of this coloration is unknown. Furthermore, dark coloured compression wood can be confused with late wood [6]. There is thus a need to devise reliable methods for detecting compression wood.

Compression wood differs from normal and opposite woods anatomically and chemically. Anatomically, the tracheids, the predominant cell type in softwoods, are shorter and more rounded in cross section in compression wood than in normal or opposite woods. The tracheid walls in compression wood are thicker, but lack the third secondary wall layer (S3) that in normal and opposite wood tracheids occurs adjacent to the lumen [5,6]. Chemically, the tracheid walls of normal and opposite woods are composed of microfibrils of cellulose set in a matrix of lignin composed predominantly of guaiacyl units (G-units), and the non-cellulosic polysaccharides (hemicelluloses) heteromannans (*O*-acetyl-galactoglucomannans) and smaller proportions of heteroxylans [arabino(4-*O*-methylglucurono)xylans] [7,8,9,10,11]. The tracheid walls of compression wood contain less cellulose, heteromannans and heteroxylans, but more lignin, which in addition to G-units contains *p*-hydroxyphenyl units (H-units) that are almost absent from normal and opposite woods. These walls also contain much higher proportions of (1→4)-β-d-galactans (up to ~10%) than normal or opposite woods [5,10,12]. All of these features of compression wood apply to what is frequently termed severe compression wood, although mild compression woods of different severities with anatomical, and possibly chemical, features intermediate between severe compression wood and normal and opposite woods are also known to occur, but have been subjected to much less research [6,13].

*Pinus radiata* (radiata pine or Monterey pine) is a fast-growing softwood widely grown in plantations in many temperate countries, including New Zealand. The compression wood of this species can be distinguished from normal and opposite woods by detailed chemical analyses of the cell-wall polymers [12,13]. The aim of the present study was to develop a method of detecting compression wood based on the occurrence of higher proportions of (1→4)-β-galactans in the tracheid walls of compression wood than normal or opposite woods. The method involves extracting non-cellulosic polysaccharides from milled wood and determining the content of (1→4)-β-galactans using an immuno-dot assay, with lupin seed (1→4)-β-galactans as the quantification standard. The extracted polysaccharides are bound to nitrocellulose membranes as dots and then treated with the monoclonal antibody LM5, which binds specifically to (1→4)-β-galactans [14], as the primary antibody. An enzyme-labelled secondary antibody is then used followed by a substrate that gives an insoluble coloured product [15]. The intensity of the colour is measured by image analysis. Essentially, we developed a simplified manual version of the technique known as comprehensive microarray polymer profiling [16,17], but with only a single extraction solvent and primary antibody.

## 2. Results

### 2.1. Assays Using Reference Lupin Seed (1→4)-β-Galactans

**Figure 1:**
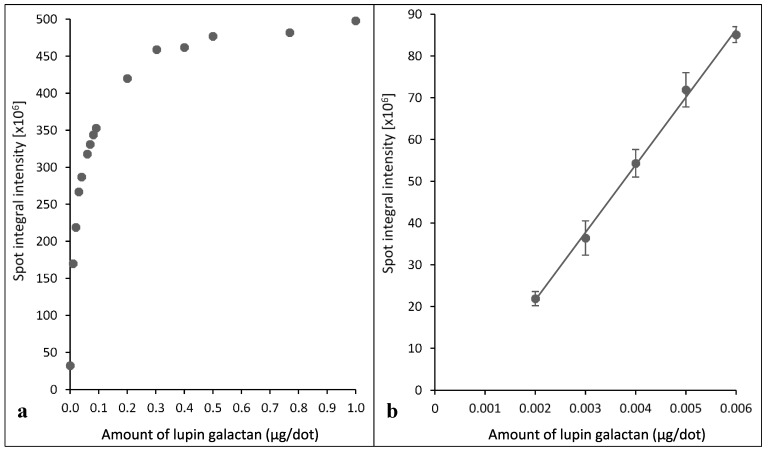
(**a**) Curvilinear relationship of spot integral intensity to amount of lupin seed (1→4)-β-galactans in the dilution series generated with Xplore software; (**b**) linear relationship of spot integral intensity to amount of lupin seed (1→4)-β-galactans at lower dilutions showing a strong positive correlation of amounts of (1→4)-β-galactans to the spot integral intensity (R^2^ = 0.99; y = (2(10^10^)x−1(10^7^)); (R^2^ = Coefficient of determination; y = spot integral intensity; x = amount of galactan (µg/dot)). Error bars indicate the standard errors.

A plot of spot integral intensities against the amounts of lupin seed (1→4)-β-galactans for a dilution series from 0.001 to 1.0 µg/dot was curvilinear (Figure 1), with the linear portion in the lower dilution range (up to 0.006 µg/dot). It is thus important to use (1→4)-β-galactan amounts in this dilution range for quantification purposes.

**Figure 2:**
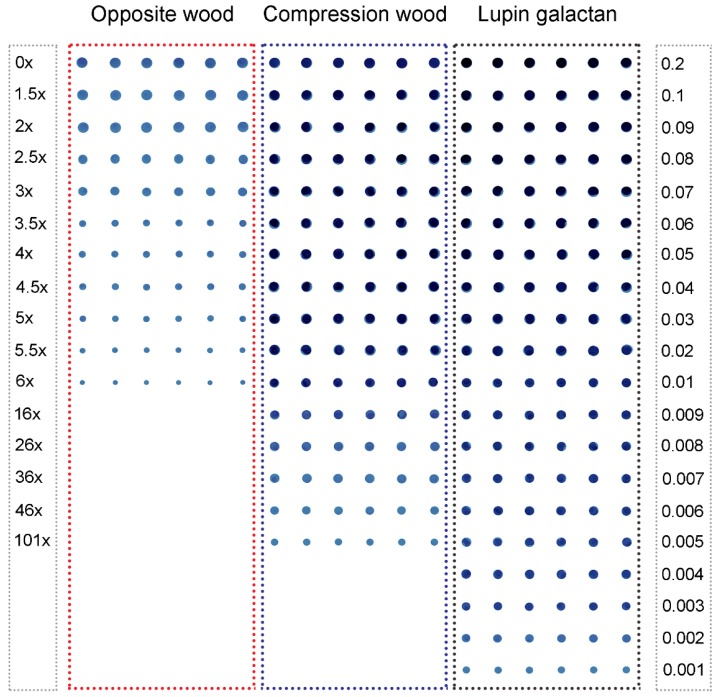
Immuno-dot assays of (1→4)-β-galactans extracted from non-delignified wood from Tree 1. Opposite wood (10 dilutions 1.5× to 6×), compression wood (15 dilutions 1.5× to 101×) and lupin seed (1→4)-β-galactan (twenty dilutions giving 0.001 to 0.2 µg/µL or dot) plotted in increasing order of dilution; each dilution has six replicates. This shows a scanned image (1200 dpi) of the nitrocellulose membrane with blue dots.

Blue dot images obtained from the dilution series of the standard lupin seed (1→4)-β-galactans are shown in Figure 2.

All lupin seed (1→4)-β-galactan amounts, from 0.001 µg/dot upwards, were readily detected by eye (Figure 2). A similar control dilution series without primary antibody showed no coloration, as did a no-(1→4)-β-galactan control dot.

### 2.2. Assays Using Extracts of Radiata Pine Wood

The dilution series of the extracts from non-delignified compression wood and opposite wood from Tree 1 (Figure 2) showed that (1→4)-β-galactans could be detected in the opposite wood extract down to only a 6× dilution, but in the compression wood extract down to a 101× dilution, indicating a higher concentration of (1→4)-β-galactans in the compression wood extract than the opposite wood extract. Quantification, using dilutions on the linear part of the curve, showed 0.03% and 0.35% for the extracted (1→4)-β-galactan content of the opposite wood and compression wood, respectively (Table 1). (The 0.03% and 0.35% values mean 0.003 and 0.035 mg (1→4)-β-galactan were extracted into the 6 M NaOH from each 10 mg of dry wood, respectively). However, when the three-year-old trees (Trees 2–7) were compared, no differences in extracted (1→4)-β-galactan contents were found between the compression wood and opposite wood of each tree (Table 1). The extracted (1→4)-β-galactan content of the opposite and compression wood was similar to that found in the opposite wood from Tree 1.

**Table 1:** Immuno-dot assay of (1→4)-β-galactans extracted from opposite and compression woods of *Pinus radiata* with and without delignification.

Tree	Amounts of (1→4)-β-Galactans Extracted Without Delignification			Amounts of (1→4)-β-Galactans Extracted after Delignification		
	OW	CW	CW/OW	OW	CW	CW/OW
	Mean ± SE	Mean ± SE		Mean ± SE	Mean ± SE	
Tree 1	0.03 ± 0.00	0.35 ± 0.03	11.7	0.38 ± 0.09	9.95 ± 1.39	26.2
Tree 2	0.02 ± 0.00	0.01 ± 0.00	0.5	0.60 ± 0.11	8.34 ± 0.60	14.0
Tree 3	0.01 ± 0.00	0.01 ± 0.00	1.0	0.42 ± 0.08	8.25 ± 0.02	19.5
Tree 4	0.03 ± 0.00	0.02 ± 0.00	0.7	0.60 ± 0.03	6.63 ± 0.95	11.1
Tree 5	0.01 ± 0.00	0.02 ± 0.00	2.0	0.39 ± 0.02	5.92 ± 0.76	15.3
Tree 6	0.03 ± 0.00	0.04 ± 0.00	1.3	0.22 ± 0.04	4.27 ± 0.50	19.5
Tree 7	0.03 ± 0.00	0.03 ± 0.00	1.0	0.74 ± 0.08	3.14 ± 0.10	4.2

OW = Opposite wood; CW = Compression wood. CW/OW = ratio of amounts of (1→4)-β-galactans extracted from compression and opposite woods. Amounts of galactans extracted are expressed as weight percentages extracted from dry wood (means of three aliquots of milled wood).

**Figure 3:**
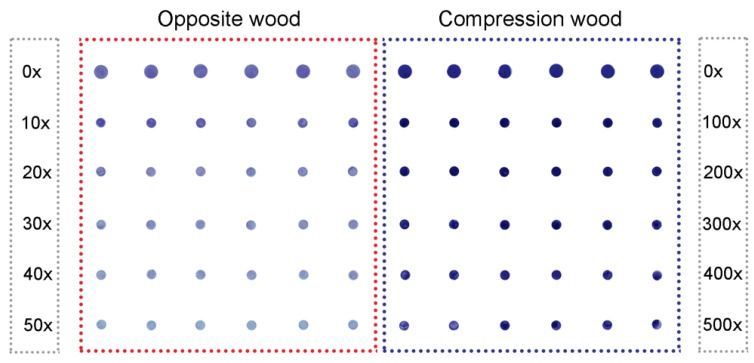
Immuno-dot assays of (1→4)-β-galactans in extracts of delignified wood of Tree 7. Extracts of opposite wood (five dilutions 0× to 50×) and compression wood (five dilutions 0× to 500×; each dilution has six replicates. This shows a scanned image (1200 dpi) of the nitrocellulose membrane with blue dots.

We therefore examined the effect of partially delignifying the milled wood on the amounts of (1→4)-β-galactans extracted by the 6 M NaOH solution. After partial delignification, much greater amounts of (1→4)-β-galactans were extracted, particularly from the compression wood. For example, with the wood from Tree 7, (1→4)-β-galactans were detected in the opposite wood extract down to only a 50× dilution, but in the compression wood extract, (1→4)-β-galactan was still detected at a 500× dilution (Figure 3). The particular dilutions were chosen because 20× to 50× for opposite wood and 200× to 500× for compression wood were on the linear part of the curve. The calculated extracted concentrations ranged from 0.22% to 0.74% for opposite wood and 3.14% to 9.95% for compression wood, with the highest concentrations of extracted (1→4)-β-galactans being in the compression wood of Tree 1 (Table 1).

**Figure 4:**
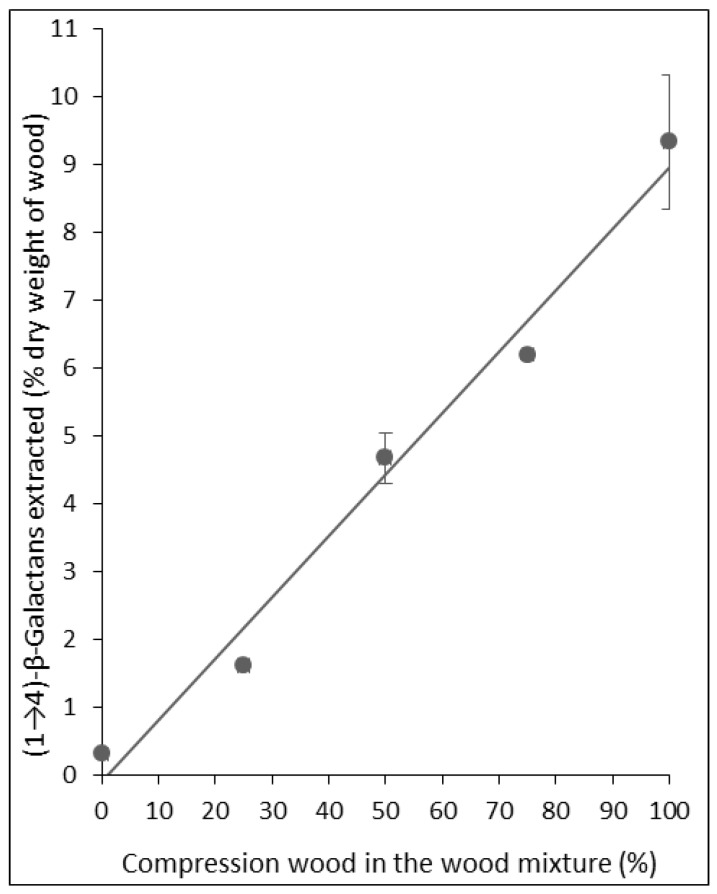
The amount of (1→4)-β-galactans extracted from delignified mixtures of compression wood and opposite wood from Tree 1 showing a linear relationship with amount of compression wood in the mixture, with a strong positive correlation (R² = 0.98; y = 0.0905x−0.0866). (R² = Coefficient of determination; y = spot integral intensity; x = amount of galactan (µg/dot)). Error bars indicate standard errors (some values have negligible standard errors, so the error bars are not visible).

The upper value for extracted (1→4)-β-galactans in opposite wood was 0.74% (Tree 7) and could represent the upper value for wood not containing any compression wood. The lowest concentration for compression wood was 3.14% (Tree 7) and could represent one of the lowest that may be obtained from severe compression wood. However, although the Tree 7 compression wood sample was dark coloured, it was not examined microscopically and may not be pure severe compression wood. It may have also contained some tracheids better described as mild compression wood tracheids, which may have extracted (1→4)-β-galactan concentrations intermediate between opposite (or normal) wood and severe compression wood [13]. We thus tested the immuno-dot assay method on mixtures of compression wood and opposite wood to simulate different mild compression woods. Such mixtures would also simulate samples containing adjacent compression and normal (or opposite) woods. As expected, the relationship between the content of extracted (1→4)-β-galactans and percentage of compression wood in the mixture was linear (Figure 4).

### 2.3. Monosaccharide Compositions of Radiata Pine Wood

To show that the compression and opposite woods examined in the present study contained different proportions of galactosyl-containing polysaccharides, the woods were acid hydrolysed and analysed. The proportions of the neutral monosaccharides in the 2 M TFA hydrolysates of the opposite and compression woods are shown in Table 2. The highest proportions of particular monosaccharides were for galactose in compression wood hydrolysates, probably mostly from (1→4)-β-galactans. The proportions ranged from 38.1% (Tree 7) to 58.5% (Tree 3). Galactose also showed the greatest differences in proportions between compression and opposite woods, with the proportions in the opposite woods ranging from 7.7% (Tree 3) to 10.5% (Trees 2 and 5). This gave ratios of the proportion of galactose in the compression wood to opposite wood ranging from 4.3 (Tree 7) to 7.6 (Tree 3). The proportions of xylose (mostly from heteroxylans) and mannose (mostly from heteromannans) were higher in hydrolysates of opposite than compression woods, giving ratio (opposite/compression) ranges of 1.8–3.4 and 1.5–2.5, respectively. A similar ratio range, 1.3–2.3, was also found for arabinose (probably mostly from heteroxylans), but the proportions were lower. In contrast, the proportions of glucose in hydrolysates of opposite and compression woods were similar, giving a ratio (opposite/compression) range of 0.8–1.2.

**Table 2:** Neutral-monosaccharide compositions of opposite and compression woods (% of all neutral monosaccharides).

	Monosaccharides						
		Arabinose	Xylose	Galactose	Glucose	Mannose	CW/OW Galactose
Tree	Wood Type	Mean ± SE	Mean ± SE	Mean ± SE	Mean ± SE	Mean ± SE	
Tree 1	OW	10.2 ± 0.53	32.2 ± 0.12	8.4 ± 0.07	13.6 ± 0.26	35.6 ± 0.24	6.3
	CW	4.7 ± 0.36	15.3 ± 0.29	52.7 ± 0.25	11.4 ± 0.11	16.0 ± 0.28	
Tree 2	OW	13.2 ± 0.23	35.8 ± 0.47	10.5 ± 0.20	9.4 ± 0.30	31.2 ± 0.18	5.5
	CW	5.8 ± 0.06	13.1 ± 0.13	56.9 ± 0.49	9.8 ± 0.23	14.3 ± 0.25	
Tree 3	OW	12.0 ± 0.33	38.7 ± 0.11	7.7 ± 0.10	10.1 ± 0.37	31.8 ± 0.46	7.6
	CW	5.3 ± 0.26	11.4 ± 0.17	58.5 ± 0.28	12.2 ± 0.27	12.6 ± 0.29	
Tree 4	OW	13.3 ± 0.35	31.4 ± 0.71	10.2 ± 0.17	12.4 ± 0.25	32.7 ± 1.03	4.9
	CW	6.5 ± 0.24	15.9 ± 0.18	49.3 ± 0.12	10.4 ± 0.34	17.8 ± 0.78	
Tree 5	OW	10.2 ± 1.44	30.4 ± 0.61	10.5 ± 0.05	13.1 ± 0.31	35.8 ± 0.89	4.6
	CW	7.6 ± 0.29	15.7 ± 0.08	47.7 ± 0.89	11.9 ± 0.16	17.3 ± 0.39	
Tree 6	OW	12.3 ± 0.21	29.5 ± 0.31	7.9 ± 0.08	12.5 ± 0.23	37.8 ± 0.19	6.8
	CW	6.8 ± 0.02	11.9 ± 0.07	53.7 ± 0.41	11.2 ± 0.29	16.5 ± 0.11	
Tree 7	OW	10.2 ± 0.90	34.4 ± 0.35	8.9 ± 0.09	11.1 ± 0.23	35.4 ± 0.29	4.3
	CW	7.4 ± 0.13	18.9 ± 0.39	38.1 ± 1.46	11.3 ± 0.29	24.4 ± 0.85	

Mean of determinations on three hydrolysates. CW/OW Galactose is the ratio of the percentage of galactose in compression wood and opposite wood.

The rankings of the compression woods of the three-year-old trees (Trees 2–7) for percentages of galactose in hydrolysates and percentages of (1→4)-β-galactans in extracts were similar; the only major difference was for Tree 6, which ranked higher in terms of percentage galactose than percentage of (1→4)-β-galactan extracted.

As with the extracted (1→4)-β-galactan concentrations, upper limits of ranges can be set for opposite wood and compression wood. The highest galactose percentage for opposite wood of 10.5% could be used as the upper limit for wood not containing compression wood. The lowest galactose percentage for compression wood, 38.1%, could be used as the lowest percentage for severe compression wood, although the compression wood for this tree may not be pure severe compression wood.

For the mixtures of compression wood and opposite wood, the relationship between percentage galactose and percentage of compression wood in the mixture was again linear [18].

## 3. Discussion

Comprehensive microarray polymer profiling and its methods for polysaccharide extractions was developed and has subsequently been used most frequently on plant tissues containing principally non-lignified primary cell walls [16,17]. However, in wood science, the non-cellulosic polysaccharides are usually extracted from wood cell walls after they have first been delignified, yielding a fraction known as holocellulose. This is because non-cellulosic polysaccharides are difficult to extract from lignified walls, particularly those of softwoods [19,20]. This is consistent with our finding that partial delignification of the milled radiata pine wood with acid chlorite [21] resulted in the subsequent extraction of more (1→4)-β-galactans. Similar (1→4)-β-galactans were also more easily extracted from the compression wood of Norway spruce (*Picea abies*) after acid chlorite delignification [22]. The original acid chlorite delignification process [21] aimed to achieve complete delignification and involves prolonged treatment with fresh charges of sodium chlorite and acetic acid being added at intervals. However, in the present study, we simplified the delignification treatment by omitting fresh charges. This was done to simplify the process, and to reduce the likelihood of the (1→4)-β-galactans being degraded or extracted during the delignification. Although the delignification is unlikely to have been complete, the process can easily be reproduced and is sufficient. Using the immuno-dot assay, the percentage of (1→4)-β-galactans extracted from the compression wood of Tree 1, 9.95%, compared well with the 10.3% for the galactose content of compression wood from a tree of the same genotype (an analogous ramet), but a different age, using a two-stage sulphuric acid hydrolysis [12]. Using the immuno-dot assay, we found only trace amounts of (1→4)-β-galactans in the acid chlorite solution after treatment of compression wood. In a study of the delignification of black spruce (*Picea mariana*) by the chlorite process [21], it was found that during the first 60% of delignification only lignin was dissolved, and beyond that point small quantities of galactoglucomannans were also dissolved [23].

The increased amounts of (1→4)-β-galactans extracted from both compression and opposite woods of radiata pine after partial delignification suggests these polysaccharides are held in the tracheid walls by being covalently linked to lignin. Lignin-carbohydrate complexes, containing such covalent bonds, have been isolated from a variety of woods [10], including a complex containing lignin and (1→4)-β-galactans from the compression wood of Japanese red pine (*Pinus densiflora*) [24]. These lignin-polysaccharide cross links are formed by reactions involving quinone methide intermediates, produced during lignin synthesis, and polysaccharides [25]. Consistent with covalent links between lignin and (1→4)-β-galactans has been the finding, using immunomicroscopy with LM5, that lignins and (1→4)-β-galactans are colocated in the tracheid walls of radiata pine. In normal and opposite woods, the small proportion of (1→4)-β-galactans found in these woods have been localized to the tracheid primary wall [26,27]. This is consistent with the (1→4)-β-galactans occurring as side chains of the pectic polysaccharide rhamnogalacturonan I (RG-1), which is known to occur in the primary cell walls of coniferous gymnosperms [12,28]. The highest concentrations of lignin in these wood types also occur in the compound middle lamella comprising the middle lamella and primary wall. In the mature tracheids of compression woods, the (1→4)-β-galactans have been localized to the outer S2 layer, a region in which the highest concentration of lignin also occurs and is hence referred to as the S2L layer. Using the same antibody, the (1→4)-β-galactans were also localized to the same layer in mature compression wood tracheids of Japanese cedar (*Cryptomeria japonica*) [29]. Interestingly, in that study, the intensity of the labelling over the S2L layer was higher after acid chlorite delignification of the wood.

The immuno-dot method could also be extended to other species of coniferous gymnosperms. It is likely to be particularly useful for species where other galactose-containing polysaccharides are known to occur in the wood because the method is specific to (1→4)-β-galactans. For example, the wood of larches (*Larix* spp.), especially the heartwood, contains high proportions of water-soluble arabino-3,6-galactans, which are non-structural polysaccharides. However, compression wood of larches also does contain (1→4)-β-galactans [30]. Lower proportions of arabino-3,6-galactans occur in the heartwoods of other coniferous gymnosperms, including Norway spruce (*Picea abies*) and Scots pine (*Pinus sylvestris*) [10,11,31,32,33]. Previous studies using 2D NMR [12] on compression and opposite wood samples from a tree of the same genotype (an analogous ramet), as Tree 1, but a different age, showed that the higher proportions of galactose in the compression wood hydrolysates were due to higher proportions of (1→4)-β-galactans; there was no evidence for the presence of arabino-3,6-galactans.

Although a TFA hydrolysis method was used in the present study to show differences in proportions of galactosyl-containing polymers between the opposite and compression woods, this method of acid hydrolysis could itself be used as a method to detect compression wood, although it is, of course, not specific for (1→4)-β-galactans. In wood science, the usual method of hydrolysing wood cell walls is the two-stage sulphuric acid method [12,34], which hydrolyses all the cell-wall polysaccharides, including cellulose. However, in studies of primary cell walls, the TFA hydrolysis method [35], which does not hydrolyse crystalline cellulose [36], is often used to study non-cellulosic polysaccharides. The TFA can be easily removed, so analyses can be carried out on very small samples, if required. We found this method of hydrolysis excellent for comparing the percentages of neutral monosaccharides from opposite and compression woods, with galactose being a high proportion of the neutral monosaccharides released from compression woods, but not from opposite woods. By simply using relative percentages of neutral monosaccharides rather than absolute yields of each monosaccharide on a wood dry weight basis, accurate weighing of the wood samples and addition of an internal quantification standard are unnecessary. We separated and quantified the neutral monosaccharides in the TFA hydrolysate using HPAEC, but these monosaccharides could also be converted to alditol acetates and separated and quantified by gas chromatography [37,38].

In contrast to HPAEC and gas chromatography, the immuno-dot method of detecting compression wood uses relatively simple equipment, but it is time consuming and labour intensive, particularly grinding of the wood. However, the comprehensive microarray polymer profiling technique [16,17], on which it is based, uses robotics. A high-throughput platform, which also includes a grinding step, has been developed for screening milligram quantities of plant biomass to determine cell-wall digestibility, and uses the iWALL robot [39]. Similar robotic techniques could be adapted for quantifying (1→4)-β-galactans in wood using the immuno-dot method. This method could then be used in wood research and to screen the wood of saplings in breeding programmes.

## 4. Experimental

### 4.1. Wood Samples

Samples of compression and opposite woods were obtained from seven saplings of *Pinus radiata* (D. Don) grown tilted at ~45° from the vertical; we refer to these as Trees 1–7. Tree 1 (clone A; 96047/98015, Arborgen Australasia, TeTeko, Whakatane, New Zealand) was grown in an unheated glasshouse at the University of Canterbury, Christchurch, New Zealand, for nine months [40] and then outside for 11 months. Trees 2–7, from six different control-pollinated families (one tree per family), were grown for three years in the field at Amberley, Canterbury, New Zealand. The families were as follows: Tree 2 (family 05–854), Tree 3 (family 99–405), Tree 4 (family 05–829), Tree 5 (family 05–862), Tree 6 (06–524) and Tree 7 (05–844) [41].

A basal segment (about ~15 cm long) was cut from each tree, debarked, cut longitudinally into compression wood and opposite wood (based on the dark coloration of the compression wood) with a band saw and chisel, and dried at 35 °C to ~4% moisture content. The wood was then milled to pass a 60-mesh screen (251 μm pore size) using a Wiley^®^ mini-mill (Thomas Scientific, Swedesboro, NJ, USA), dried at 105 °C and stored over silica gel. Mixtures of milled compression wood and opposite wood (75, 50 and 25% w/w compression wood) were also prepared from Tree 1 to simulate samples containing adjacent compression and normal (or opposite) woods or mild compression woods of different severities [42].

### 4.2. Extraction of Non-Cellulosic Polysaccharides from the Wood Samples

Milled wood was extracted either directly or after partial delignification that was done as follows. Milled wood (10 mg) was treated with a freshly-prepared aqueous mixture of sodium chlorite (1% w/v) (1 mL) and 17.4 M acetic acid (20 µL) for 3 h at 70 °C using a Thermomixer Comfort (Eppendorf AG, Hamburg, Germany) operated at 1400 rpm [21]. After cooling to room temperature, the mixture was centrifuged (15,700× g, 10 min), the supernatant removed, and the pellet washed (3×) with water (1 mL) followed by centrifugation (5 min). The remaining holocellulose preparation or dry milled wood (10 mg) was extracted on an orbital shaker at 180 rpm for 16 h at 37 °C with 6 M NaOH containing 1% NaBH_4_ (500 μL) to prevent alkaline peeling. After centrifuging (10 min), the supernatant was removed and neutralized with 17.4 M acetic acid, using a 2:1 (v/v) ratio of supernatant to acetic acid to achieve a pH >5 <6. A dilution series was prepared from each of these extracts using water. These dilutions are referred to as × dilutions; for example a 1.5× dilution was prepared by adding 0.5 volumes of water to a 1.0 volume of extract.

### 4.3. Immuno-Dot Quantification of (1→4)-β-Galactans

Six replicates (1 μL) of each successive extract dilution were applied as dots to a Protran^®^ nitrocellulose membrane (0.45 μm pore size; Schleicher & Schuell BioScience, Dassel, Germany) and dried at 30 °C for 1 h [16,17]. The membrane was incubated in phosphate-buffered saline (PBS) (0.01 M sodium phosphate buffer, pH 7.4; 0.14 M NaCl) containing 5% (w/v) milk powder (0.1% fat; Alpine, Dairyworks Ltd, Christchurch, New Zealand) (MP-PBS) for 2 h at 30 °C to block all nonspecific binding sites. It was then incubated with the monoclonal antibody LM5 (PlantProbes, Leeds, UK) diluted 1:10 in MP-PBS, followed by goat anti-rat IgG (H + L chains) secondary antibody conjugated with alkaline phosphatase (Invitrogen, Auckland, New Zealand) diluted 1:100 in MP-PBS. Both antibodies were incubated for 2 h at 30 °C, and after each incubation, the membrane was washed (5×) with PBS on an orbital shaker (600 rpm). Finally, the membrane was treated for 15 min with substrate solution containing 5-bromo-4-chloro-3-indolyl phosphate (BCIP), nitro-blue tetrazolium (NBT) and water (1:1:8 v/v) (BCIP/NBT substrate kit, Invitrogen, New Zealand). Alkaline phosphatase reacts with the substrate to produce an insoluble blue product. The membrane was then washed in running water for 20 min and dried at 30 °C. Two types of negative control were used: a no primary antibody control and a no extract control in which 6 M NaOH (containing 1% NaBH_4_) after the addition of acetic acid (see above) was used.

The dried nitrocellulose membranes were scanned to produce TIFF images (1200 dpi) using a desktop scanner (Model DCP-130C; Brother International, Bridgewater, NJ, USA) that were then converted into inverted 16-bit grey-scale images using Adobe Photoshop (Creative Suite 6; Adobe Systems, San Jose, CA, USA). Xplore image processing software (LabNEXT Inc, West New York, NJ, USA), which includes grids, was then used to generate spot integral intensities (heat map values) of the spots, which were exported as comma separated values (csv) and opened in Excel format. The spot integral intensity is a specific numerical value generated by the Xplore program and is based on the numbers of spots per unit area defined by a grid. Mean values for each set of six replicate spot integral intensities were computed. The final spot integral intensities were calculated by subtracting the values of blank (no sample) spots. These were converted into mg of (1→4)-β-galactans extracted per 100 mg of dry wood (*i.e.*, weight percentages) by comparing the intensities with those given by the lower range (0–0.006 µg/dot) (Figure 1) of a dilution series of lupin seed (1→4)-β-galactans. In the final calculation, it was assumed that the lupin seed (1→4)-β-galactans preparation contained 91% (1→4)-β-galactan as indicated in the Megazyme literature.

### 4.4. Neutral Monosaccharide Compositions of the Wood Samples

Triplicate samples (5 mg) of dry milled opposite and compression woods were hydrolysed using 2 M trifluoroacetic acid (TFA) (0.5 mL, 121 °C, 1 h) in a sealed tube under argon [35,38]. After cooling, d-ribose (500 µg) was added as a retention time standard, and the hydrolysate evaporated to dryness in a stream of air. The residue was dissolved in water (5 mL), filtered using a polytetrafluoroethylene disposable filter (diameter 13 mm, pore size 0.22 µm; Shandong Hapool Medical Technology, Heze, China), and the filtrate made up to 50 mL.

Neutral monosaccharides were separated and quantified using high-performance anion-exchange chromatography with pulsed amperometric detection (HPAEC-PAD) on a Dionex BioLC system (Dionex, Sunnyvale, CA, USA) fitted with an ED50 electrochemical detector and GP50 gradient pump. A CarboPac PA20 guard column (3 × 30 mm) and a CarboPac PA20 analytical column (3 × 150 mm) were used. Column temperature was kept at 25 °C by a TCC-100 thermostatted column compartment. Separation was achieved using isocratic elution (1 mM NaOH for 30 min) and the column was then washed for 5 min with 200 mM NaOH before equilibration for 5 min with 1 mM NaOH. The injection volume was 20 µL, and the flow rate 0.4 mL min^−1^. The order of elution of monosaccharides was confirmed by running solutions of individual reference monosaccharide. Before the hydrolysate runs, a water blank was run followed by a reference solution containing 10 µg mL^−1^ each of l-arabinose, d-galactose, d-glucose, d-xylose, d-mannose, d-xylose and d-ribose, which was used to determine the relative responses of equal weights of each monosaccharide.

## 5. Conclusions

The present study shows that the immuno-dot assay method using the LM5 antibody can be applied, after partial delignification, to milled (ground) wood of radiata pine for the quantification of (1→4)-β-galactans. The percentages of extracted (1→4)-β-galactans from opposite wood and compression wood were quite different and the method could thus be used to identify the presence of compression wood in wood samples. The present study is a proof of principle, but more extensive studies need to be carried out to determine the range in percentages of extracted (1→4)-β-galactans in microscopically characterized compression woods, both severe and mild, as well as normal and opposite woods, in trees of different species, genotypes and ages.
